# Amino Acid Signatures to Evaluate the Beneficial Effects of Weight Loss

**DOI:** 10.1155/2017/6490473

**Published:** 2017-04-16

**Authors:** Nina Geidenstam, Martin Magnusson, Anders P. H. Danielsson, Robert E. Gerszten, Thomas J. Wang, Lovisa E. Reinius, Hindrik Mulder, Olle Melander, Martin Ridderstråle

**Affiliations:** ^1^Department of Clinical Sciences Malmö, Clinical Obesity, Lund University Diabetes Center, Lund University, Malmö, Sweden; ^2^Department of Cardiology, Skåne University Hospital, Malmö, Sweden; ^3^Department of Clinical Sciences Malmö, Lund University, Malmö, Sweden; ^4^Cardiology Division, Massachusetts General Hospital, Harvard Medical School, Boston, MA, USA; ^5^Cardiovascular Research Center, Massachusetts General Hospital, Harvard Medical School, Boston, MA, USA; ^6^Division of Cardiovascular Medicine, Vanderbilt University Medical Center, Nashville, TN, USA; ^7^Department of Biosciences and Nutrition, Center for Innovative Medicine, Karolinska Institute, Stockholm, Sweden; ^8^Department of Clinical Sciences Malmö, Molecular Metabolism, Lund University Diabetes Center, Lund University, Malmö, Sweden; ^9^Center of Emergency Medicine, Skåne University Hospital, Malmö, Sweden; ^10^Steno Diabetes Center A/S, Gentofte, Denmark

## Abstract

*Aims*. We investigated the relationship between circulating amino acid levels and obesity; to what extent weight loss followed by weight maintenance can correct amino acid abnormalities; and whether amino acids are related to weight loss. *Methods*. Amino acids associated with waist circumference (WC) and BMI were studied in 804 participants from the Malmö Diet and Cancer Cardiovascular Cohort (MDC-CC). Changes in amino acid levels were analyzed after weight loss and weight maintenance in 12 obese subjects and evaluated in a replication cohort (*n* = 83). *Results*. Out of the eight identified BMI-associated amino acids from the MDC-CC, alanine, isoleucine, tyrosine, phenylalanine, and glutamate decreased after weight loss, while asparagine increased after weight maintenance. These changes were validated in the replication cohort. Scores that were constructed based on obesity-associated amino acids and known risk factors decreased in the ≥10% weight loss group with an associated change in BMI (*R*^2^ = 0.16–0.22, *p* < 0.002), whereas the scores increased in the <10% weight loss group (*p* < 0.0004). *Conclusions*. Weight loss followed by weight maintenance leads to differential changes in amino acid levels associated with obesity. Treatment modifiable scores based on epidemiological and interventional data may be used to evaluate the potential metabolic benefit of weight loss.

## 1. Introduction

Pathophysiological changes developing in the presence of obesity are often present long before the chronic hyperglycemia of type 2 diabetes (T2D) is manifest [1]. During these early stages, T2D can often be prevented by lifestyle changes, including weight loss and increased physical activity [2, 3]. More substantial weight loss achieved by bariatric surgery may even lead to remission of manifest T2D [4–6]. However, bariatric surgery is neither suitable nor available for all patients who may benefit from it. Therefore, early detection of subjects at risk and eligible for less invasive preventive measures is of great importance.

Predictors such as heritability, body mass index (BMI), waist circumference (WC), and fasting plasma glucose are helpful in gauging T2D risk, and in the recent years, metabolic profiling of phenotypic abnormalities have provided additional information [7–10]. Several circulating metabolites, which may reflect the biochemical environment of an individual, have shown altered levels in metabolic disorders. Notably, the aromatic and branched-chain amino acids (BCAAs) have been associated with, as well as predict, the development of insulin resistance [11, 12]. They have also been associated with the risk of developing T2D, and a combination of isoleucine, tyrosine, and phenylalanine was a particularly strong predictor of incident T2D [13, 14]. We extended this finding by demonstrating that the same amino acids (AAs) are associated with risk of future cardiovascular disease (CVD) even after adjusting for insulin resistance and T2D [15].

Recently, there has been an increased focus on metabolite levels following weight loss intervention in order to resolve which biological factors change in response to reduced weight and improved health profile [16–24]. For instance, BCAAs were found to predict improvement in insulin resistance with moderate weight loss [19]. However, the potential weight loss-associated improvements of the BCAAs have been inconsistent and further analysis is required [18–24].

In this report, we investigated the association between plasma AA levels and measures of obesity, WC, and BMI in a large population-based study to identify AAs independently associated with obesity. We then profiled AA changes during a weight loss intervention followed by a period of weight maintenance, allowing detection of when specific weight loss-related changes may occur for obesity-associated AAs. Scores were constructed that potentially can be used as predictors of the benefit of pursuing a weight loss and weight maintenance program, that is, identifying subjects that are not only at risk because of obesity but also likely to benefit from weight loss. Finally, we included a replication cohort to further evaluate weight loss-induced changes in AA levels and risk scores.

## 2. Methods

### 2.1. Study Cohorts

#### 2.1.1. The Malmö Diet and Cancer Cardiovascular Cohort (MDC-CC)

The Malmö Diet and Cancer (MDC) study is a population-based, prospective epidemiologic cohort of 28,449 persons enrolled between 1991 and 1996 in Southern Sweden. From this cohort, 6103 persons were randomly selected to participate in the MDC Cardiovascular Cohort (MDC-CC), which was originally designed to investigate the epidemiology of carotid artery disease [25, 26]. The study subjects with metabolomics data were derived from a nested incident CVD case-control study (*n* = 506) with subjects matched for gender, age, and the Framingham risk score [27] and a nested incident diabetes case-control study (*n* = 326) originating from the MDC-CC as described previously [15]. Subjects who had participated in both studies above were excluded (*n* = 27), leaving 805 individuals, 804 out of whom had complete data on all covariates and were used in this report (Table 1).

#### 2.1.2. Weight Loss and Weight Maintenance (WLWM) Cohort

To evaluate metabolite levels modifiable with a controlled weight loss intervention, 12 obese individuals referred to the Obesity Unit at Skåne University Hospital, Malmö, Sweden, and participated in a WLWM program. The intervention study has been described in detail previously (Table 1) and is independent of the MDC-CC study [7, 14, 28]. Briefly, the participants were prescribed a low-calorie diet (1200 kcal/day) for approximately three months (101 ± 26 days) followed by a six-month weight maintenance program (167 ± 37 days) consisting of group sessions with exercise and diet counseling [28]. Fasting blood samples were drawn at three time points: at baseline, after weight loss, and after weight maintenance. Subjects were weight stable at baseline and weight maintenance [28]. All subjects met the inclusion criteria of at least 10% weight loss and maintained this weight (±5%) during the weight maintenance phase.

#### 2.1.3. Replication Cohort

In order to evaluate findings from the WLWM cohort, a replication cohort was included consisting of a larger group of 83 obese individuals that participated in nonsurgical weight loss programs at the Department of Endocrinology, Skåne University Hospital, Malmö, Sweden. The programs were based on behavioral therapy (individually or in groups) and whenever possible proceeded by a prolonged period with a low-calorie diet as previously described [7, 28, 29]. Participants were included based on complete data on amino acids, both before and after weight loss treatment, as well as information regarding hypertension and T2D status. Anthropometric data are presented in Table 1. There was no overlap of subjects participating either in both the WLWM and the replication cohort or in the MDC-CC study. The study protocols were approved by the Institutional Review Boards of Lund University, Sweden. All participants provided written informed consent. Neither the WLWM nor the replication cohorts were registered as clinical trials as recruitment for these studies started in 2004, when registration was not required.

### 2.2. Clinical Assessment

Blood pressure was obtained after 10 minutes of rest in supine position. Hypertension was defined as systolic blood pressure (SBP) ≥140 mmHg or diastolic blood pressure (DBP) ≥90 mmHg, or use of antihypertensive treatment (AHT). Diabetes was defined as fasting whole blood glucose > 6.0 mmol/L, a self-reported physician diagnosis of diabetes, or use of antidiabetic medication. Fasting insulin, triglycerides, and total- and HDL cholesterol were measured according to the standard procedures at the Department of Clinical Chemistry, Skåne University Hospital, Malmö, Sweden. LDL cholesterol was calculated using the Friedewald formula. HOMA-IR was used as an estimate of insulin resistance [30].

### 2.3. Metabolite Profiling

Plasma samples were collected at baseline exam in the MDC-CC and metabolites were profiled using liquid chromatography-tandem mass spectrometry (LC-MS) as described in detail previously [14, 15, 27]. For the WLWM cohort, metabolite profiling was performed on three aliquots of 100 *μ*L plasma from each subject, and for the replication cohort, 40 *μ*L serum was analyzed. Metabolite separation and detection for both WLWM and replication cohorts were performed using gas chromatography-mass spectrometry (GC-MS) as previously described [7, 31]. In the WLWM cohort, a total of 108 plasma samples were run in randomized order. In the replication cohort, a total of 168 samples were analyzed in two randomly assembled batches. Isoleucine and leucine were detected as separate peaks in the MDC-CC analysis and replication cohort, and as one peak in the WLWM analysis, thus isoleucine and leucine will be referred as isoleucine/leucine in the WLWM cohort.

### 2.4. Data Analysis

**Figure 1:**
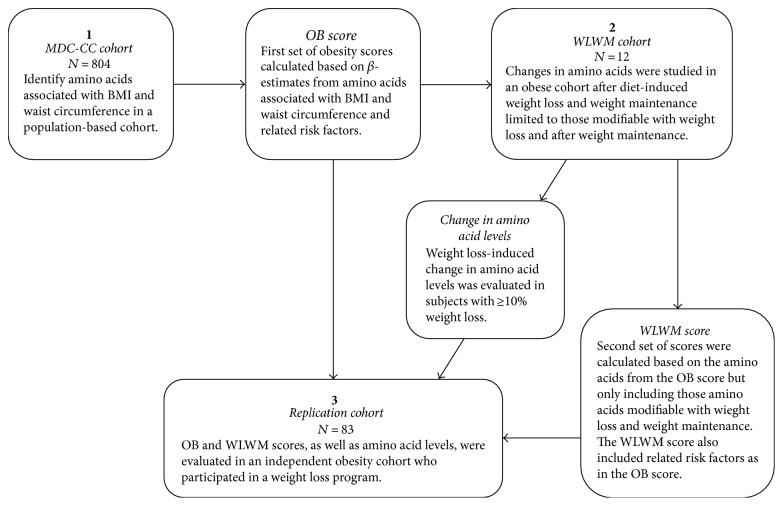
An overview of the study design. MDC-CC: Malmö Diet and Cancer Cardiovascular Cohort, OB: obesity, WLWM: weight loss weight maintenance.

The data analysis was comprised of three cohort stages (Figure 1). First, we aimed to identify AAs independently associated with continuous or categorical measures of obesity in the MDC-CC database (WC and BMI; abdominal obesity (WC > 102 cm for men; >88 cm for women [32]) and general obesity (BMI > 30 kg/m^2^)), to create AA profile scores for obesity (OB), OB-WC and OB-BMI, that included AAs positively or negatively associated with WC and BMI, respectively, along with age, gender, AHT, SBP, and T2D. Second, new scores were created using the same set of variables minus those AAs that did not show change towards levels that had been previously observed in lean insulin-sensitive individuals [8, 32–34], which suggests improvement as a result of the WLWM intervention, WLWM-WC and WLWM-BMI. The OB scores assess the total AA-associated burden of obesity, whereas the WLWM scores assess the portion of this burden which may change by a weight loss and weight maintenance program. Third, we evaluated whether these scores were associated with weight loss as well as validation of change in AA levels in an independent replication cohort.

#### 2.4.1. Amino Acid Levels Associated with Waist Circumference and BMI

In the MDC-CC, all plasma AA levels were log transformed and scaled to multiples of one standard deviation (SD). The previously reported diabetes-predictive amino acid (DM-AA) score, was modeled according to the formula: *z*-score of log *X*_1_ + *z*-score of log *X*_2_ + *z*-score of log *X*_3_ with *X_j_* denoting the AAs, isoleucine, tyrosine, and phenylalanine [14, 15]. The score was then scaled to multiples of one SD. Correlations with WC and BMI were calculated from linear regression while adjusting for age, sex, diabetes status, AHT, and SBT. Associations were considered significant at a two-tailed *p* < 0.003 (*p* < 0.05 Bonferroni corrected for the 18 measured AAs). The OB-WC and OB-BMI scores were constructed from the AA levels weighted by the *β*-coefficients calculated from backward elimination regression of all AAs which correlated to WC or BMI, respectively, adjusted for age, sex, diabetes status, AHT, and SBP. All calculations in the MDC-CC were performed in SPSS Windows (v. 20.0) or STATA (v. 12, StataCorp.).

#### 2.4.2. Changed Amino Acid Levels during the Weight Loss and Weight Maintenance Program

For the WLWM cohort, the raw data pretreatment and peak identification were performed as described previously [31]. All modeling was performed in SIMCA 13.0 (Umetrics, Umeå, Sweden), using principal component analysis (PCA) [35] or orthogonal projections to latent structures discriminant analysis (OPLS-DA) [36]. Prior to analysis, multiple peaks stemming from the same metabolite were added. Score vector from the first principal component of noncentered PCA model for isotope-labeled internal standards was used to normalize the metabolite dataset [37]. Samples were extracted and processed in triplicates so an average level was calculated prior to further analysis. Finally, the metabolite dataset was combined with hormone, cholesterol, and triglyceride levels to form the final dataset of 59 measured variables in 36 samples. Variables deviating from the normal distribution were log transformed and all variables were double centered and scaled to unit variance [38, 39]. Outliers were eliminated using a PCA model and Hotelling's *t*-test [40]. The metabolic regulation during weight loss was studied by setting up an OPLS-DA model with only the levels measured at baseline and weight loss. In the same manner, a model was calculated for weight maintenance, comparing the levels measured at weight loss and weight maintenance, and for the whole course of the program, comparing levels measured at baseline and weight maintenance. In all models, noise was reduced by limiting the number of latent components with cross-validation [41, 42]. The profile for each variable during the weight loss and weight maintenance program was constructed from the loadings from each model.

The WLWM-WC and WLWM-BMI scores were constructed similarly to the OB scores from the MDC-CC, but only included *β*-coefficients of AAs whose change, during the weight loss and weight maintenance program, moved towards levels that have previously been observed in lean subjects (judged from baseline to weight maintenance). The z-scores of the logarithmic values of each amino acid level were used. For backward elimination, an exclusion threshold of *p* < 0.100 was used. We also assessed whether the risk for CVD and T2D, represented by the previously presented DM-AA score [14, 15], could decrease by the weight loss and weight maintenance intervention in an analogous manner.

Each score (OB-BMI, OB-WC, WLWM-BMI, and WLWM-WC) was evaluated in the replication cohort. The respective AAs were log transformed and standardized before calculating the OB-BMI/WC, WLWM-BMI/WC, and DM-AA scores according to the formulas developed or previously reported. T2D cases (*n* = 17) were excluded prior to calculating the DM-AA score in replication cohort.

## 3. Results

### 3.1. Amino Acid Levels and Their Association to Measures of Obesity

Univariate linear regression analysis of data from the MDC-CC showed that the plasma levels of 12 out of 18 identified AAs were associated with WC after adjusting for sex and age (Table 2). Adjustment for T2D and CVD risk factors (SBP and AHT) reduced the number of associated AAs to ten, six showing a positive and four showing a negative correlation. With the exception of leucine, which showed no correlation to BMI in the fully adjusted model, the same AAs were associated with BMI as with WC (Table 2). The DM-AA score also showed a strong positive correlation with both WC and BMI. A significant gender effect was observed for asparagine (BMI: *β* = −0.743, *p* = 0.017, WC: *β* = −1.577, *p* = 0.047), glutamate (BMI: *β* = 0.807, *p* = 0.011, WC: *β* = 2.514, *p* = 0.002), proline (BMI: *β* = 1.091, *p* = 0.001, WC: *β* = 2.853, *p* = 0.001), and valine (only WC: *β* = 1.883, *p* = 0.025) for their association with obesity in MDC-CC. Next, we tested the association between AAs or the DM-AA score, and categorical measures of obesity such as abdominal obesity based on WC, and general obesity based on BMI (Supplementary Figure 1 available online at https://doi.org/10.1155/2017/6490473). The same AAs were significantly associated with these measures; in both cases, their odds ratios were similar, and levels of these AAs had also been associated with both WC and BMI in the preceding analysis.

To identify AAs that were independently associated with WC and BMI, we performed backward elimination regression with sex, age, SBP, AHT, and T2D status as covariates (Table 3). With WC as response variable, isoleucine and phenylalanine were eliminated, and with BMI as response variable, glutamate and phenylalanine were eliminated. The coefficient of determination for the independent association of AAs with WC was higher than for BMI (*R*^2^ = 0.54 and *R*^2^ = 0.31, resp.). From the associated AAs, we constructed two scores, OB-WC and OB-BMI, according to the following formulas signifying the relationship between obesity and AA levels:

(1) $$\begin{array}{c} OB-WC=108+2.39\left[Ala\right]-4.01\left[Asn\right]+0.96\left[Glu\right]-1.41\left[Gly\right]+2.96\left[Tyr\right]+1.21\left[Val\right]-13.4\left[sex\right]-0.12\left[age\right]+0.05\left[SBP\right]+2.27\left[AHT\right]+4.26\left[T2D\right],\\ OB-BMI=26.8+0.92\left[Ala\right]-1.40\left[Asn\right]-0.58\left[Gly\right]-0.61\left[Ile\right]+1.03\left[Tyr\right]+1.01\left[Val\right]+0.12\left[sex\right]-0.061\left[age\right]+0.02\left[SBP\right]+0.98\left[AHT\right]+1.50\left[T2D\right]. \end{array}$$

### 3.2. The Effect of Weight Loss Followed by Weight Maintenance on Amino Acids and the Obesity Scores

All participants in the controlled weight loss and weight maintenance program met the inclusion criteria of ≥10% weight loss with an average weight loss of 20.1 ± 10.2% (mean ± SD) during the weight loss phase and maintained the reduced weight (±3.9%) during the six-month weight maintenance phase (Table 1).

The PCA of the complete dataset from the weight loss and weight maintenance program showed three clusters, reflecting baseline, weight loss, and weight maintenance, suggesting that there were differences in metabolite levels between the time points (Supplementary Figure 2A). To discriminate between baseline, weight loss, and weight maintenance, an OPLS-DA model was created. The resulting score plot revealed a clear separation between the different time points, confirming that there were differences in metabolite levels between baseline, weight loss, and weight maintenance (Supplementary Figure 2B). Most AA levels (78%) changed during the weight loss intervention and 86% out of those, 67% of the 18 identified AAs, remained changed after weight maintenance. We grouped the AAs and the DM-AA, OB-BMI, and OB-WC scores according to their weight loss- and weight maintenance-change pattern into five distinct groups (Table 4). Three change patterns towards levels found in normal weight subjects were found for AA levels that showed long-term changes: (i) decrease during weight loss and sustained levels during weight maintenance, glutamate, isoleucine/leucine, serine, and the OB-WC score; (ii) decrease during weight loss followed by partial increase during weight maintenance, alanine, aspartate, aromatic AAs, tryptophan, and the DM-AA and OB-BMI scores; and (iii) no change during weight loss followed by an increase during weight maintenance, asparagine, glutamine, and methionine [8–10, 32–34, 43]. Absence of long-term changes was found for seven AAs, including glycine and the BCAA valine (Table 4).

We then performed a backward elimination regression using only the AAs, whose levels either increased or decreased as a result of the whole weight loss and weight maintenance program, that is, the modifiable part of the original OB scores, to construct weight loss and weight maintenance scores (Table 3):

(2) $$\begin{array}{c} WLWM-WC=112+2.56\left[Ala\right]-4.52\left[Asn\right]+1.04\left[Glu\right]+3.56\left[Tyr\right]-14.8\left[sex\right]-0.15\left[age\right]+0.05\left[SBP\right]+2.48\left[AHT\right]+5.27\left[T2D\right],\\ WLWM-BMI=27.8+0.93\left[Ala\right]-1.64\left[Asn\right]+0.16\left[Ile\right]+1.28\left[Tyr\right]-0.08\left[sex\right]-0.08\left[age\right]+0.02\left[SBP\right]+1.04\left[AHT\right]+1.71\left[T2D\right]. \end{array}$$

As expected, the weight loss and weight maintenance scores decreased during weight loss and sustained decreased during the weight maintenance phase (Table 4).

### 3.3. Evaluation of Weight Loss-Induced Amino Acid Changes and Scores

**Figure 2:**
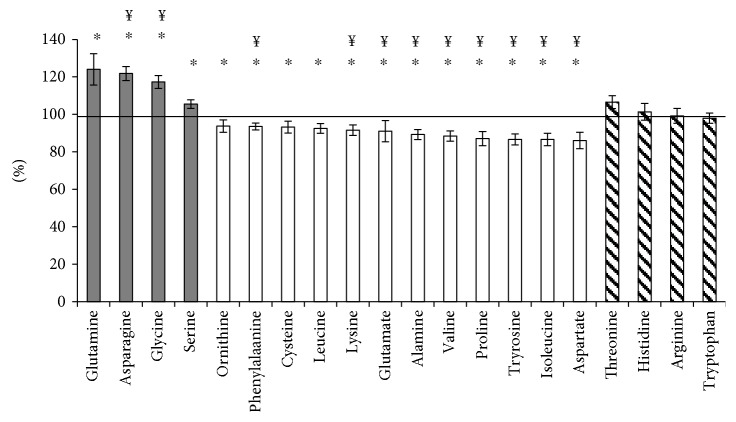
Change in amino acid levels after weight loss treatment in the replication cohort. Percentage increase (dark grey), decrease (white), and unchanged levels (striped) from the baseline to follow-up in the replication cohort are illustrated for those with ≥10% weight loss (*n* = 53). Each bar represents the change at follow-up from baseline, which was set at 100%. ^∗^*p* < 0.05, ^¥^significant after Bonferroni correction.

In order to further evaluate AA changes after weight loss treatment, we included a replication cohort with subjects that participated in a similar weight loss treatment program as the WLWM cohort except less controlled. This meant fewer clinical visits, not meeting the criterion of at least 10% weight loss, and sample collection at the visit before and after attending the weight loss program only. Thus, participants in the WLWM cohort were analyzed at baseline, immediately after weight loss, and after a weight stability phase, whereas the replication cohort was analyzed at baseline and after 345 ± 195 days, that is, baseline and weight maintenance phases in WLWM were comparable to baseline and follow-up in the replication cohort. For subjects with ≥10% weight loss (*n* = 53), an increase of 5–24% was observed for glutamine, asparagine, glycine, and serine and a decrease of 6–14% was observed for ornithine, cysteine, lysine, glutamate, alanine, proline, aspartate, and the aromatic AAs and BCAAs (Figure 2). Unchanged levels were found for threonine, histidine, arginine, and tryptophan. In total, 17 AAs were identified both in the WLWM and the replication cohort: the change from baseline to weight maintenance/follow-up could be validated for glutamine, asparagine, phenylalanine, leucine, glutamate, alanine, tyrosine, isoleucine, aspartate, and threonine.

Overall, evaluation of the obesity scores in the replication cohort (*n* = 83) showed no significant change from baseline to follow-up (Supplementary Table 1). Although, the regression of change in BMI on change in score level was strongly significant for each score (Supplementary Figure 3A–D). Baseline score levels were not associated with change in BMI from baseline to follow-up, and the scores were not different in those with ≥10% compared to <10% weight loss. When regarding those with ≥ or <10% weight loss separately, we found that the OB and WLWM scores decreased significantly in those with ≥10% weight loss (*p* < 0.003), whereas significant increases were seen in those with <10% weight loss (*p* < 0.0004). In addition, the weight loss-induced changes in both scores (OB and WLWM) were associated with change in BMI in those with ≥10% weight loss (*R*^2^ for OB-WC = 0.22, WLWM-WC = 0.17, OB-BMI = 0.19, and WLWM-BMI = 0.16; *p* < 0.002), but not in the <10% weight loss group (data not shown). The change in scores was highly significantly different (*p* < 2e10^−6^) between the two weight loss groups (≥ or <10% weight loss).

Participants with T2D were excluded in the analysis of the DM-AA score (whole cohort: *n* = 67, ≥10% weight loss: *n* = 44, <10% weight loss: *n* = 23). The DM-AA score decreased from baseline to follow-up in the group with ≥10% weight loss (*p* = 0.04), and increased in the <10% weight loss group (*p* = 0.04). The DM-AA scores in the two weight loss groups (≥ or <10%) were different at follow-up (*p* = 0.002) as was the change in DM-AA score from baseline to follow-up (*p* = 0.009), but not at baseline. This is further illustrated in scatter plots depicting change in BMI against change in DM-AA score using all nondiabetic participants (Supplementary Figure 3 E). At baseline, there was no correlation between BMI and DM-AA score, but at follow-up, and especially the change from baseline to follow-up, these were correlated (*R*^2^ = 0.16, *p* = 0.0006).

## 4. Discussion

Altered circulating AA levels have been observed in metabolic disorders, like obesity, T2D, and other insulin-resistant states since many years and recent reports have supported these findings [7–14, 19, 33, 44]. In the present study, we found a panel of eight AAs: alanine, asparagine, glutamate, glycine, phenylalanine, tyrosine, and the BCAAs isoleucine and valine, to be independently associated with obesity after adjusting for sex, age, SBP, AHT, and T2D. We included a small cohort of subjects participating in a weight loss and weight maintenance program showing changes towards levels found in normal weight subjects (by either increased or decreased plasma levels) in twelve AAs. To further validate the changes observed, or lack of change after the weight loss program, we included a replication cohort, where changes in 10 of the 17 AAs that could be compared were replicated. Findings concerning these AAs and metabolic disease are becoming increasingly robust, but the underlying mechanisms remain largely elusive.

Previous studies using metabolomics to analyze AAs levels have reported elevated levels of BCAAs in obese individuals (both with and without T2D) and in lean individuals with T2D, compared to the levels found in lean healthy subjects [8]. While the biological mechanism behind this is unclear, several reports have observed that metabolic imbalance due to constant energy overload alters not only glucose levels but also AA and fatty acid metabolism in peripheral tissues [45, 46]. Specifically, it has been suggested that elevated circulating BCAAs may be due to a deficiency of the BCAA-catabolizing enzymes in the adipose tissue of obese subjects [47–49]. Here, we investigated the associations between 18 amino acids and different measures of obesity and found a positive association for levels of alanine, glutamate, isoleucine, phenylalanine, tyrosine, and valine and a negative association for those of asparagine and glycine (adjusting for sex, age, SBP, AHT, and T2D). Only five AAs, arginine, lysine, methionine, proline, and tryptophan, showed no association in any of the models tested. The association between AA levels and obesity appears to be very robust, and we confirm the positive associations of alanine, glutamate, isoleucine, phenylalanine, tyrosine, and valine to obesity previously reported [9, 10, 32, 33]. Interestingly, leucine showed association to WC and BMI when adjusted for sex and age only, but the association was lost with additional adjustment for cardiovascular risk factors and T2D status. This may indicate a stronger association to cardiovascular risk factors and T2D than to obesity, as compared to the other two BCAAs, isoleucine, and valine. The inverse association of glycine and asparagine observed in this study confirms previous findings [32, 33, 50].

In this report, we aimed to identify AAs that are associated with obesity and to identify which AAs that change with sustained weight loss towards levels found in healthy subjects [8, 9, 32–34]. In order to do this, we first examined AA changes in a controlled weight loss and weight maintenance group. The changes in AA levels were then analyzed in a replication cohort. Levels of several AAs associated with insulin resistance have been found to change by either decreased or increased levels towards levels found in subjects with normal weight after weight loss, but few studies have included data on long-term effects [16, 18, 19, 21, 22]. While metabolite levels at baseline can predict future outcome of obesity-related metabolic disorders [12, 14, 15], multiple measurements of metabolite levels may be necessary to evaluate change in metabolic risk over time. Therefore, we also investigated which metabolite levels change with a weight loss and weight maintenance program, metabolites that return to baseline levels after weight maintenance, and metabolites that appear to be unaffected although associated by obesity. For instance, we found a decrease in BCAA levels during weight loss, parallel to the improvement in insulin resistance, as judged by HOMA-IR, and of triglyceride levels, which is in agreement with previous studies, and as validated in the replication cohort [19, 23]. However, whereas the decrease in isoleucine and leucine levels was sustained after weight maintenance, valine levels returned to baseline. Previous studies both confirm [18, 19, 21, 23] and contradict the observation [20, 21, 24] of decreased isoleucine and leucine levels below baseline levels after weight loss and weight maintenance. Here, the decrease in valine remained after sustained weight loss in our replication cohort. Differences in the amount of weight loss, cohort size, or the observation times may account for these differences between studies.

We also confirm that weight loss induces reductions of alanine, glutamate, phenylalanine and tyrosine levels in both the WLWM and replication cohort [18, 24], and show that these levels remain reduced after weight maintenance. By contrast, a recent study by Tochikubo et al. reported that unchanged levels of these AAs after weight loss were observed in both female and male subjects [23]. Since they applied a weight loss limit of 3%, this may indicate that more profound weight loss is required to observe a decrease of these amino acids. Asparagine provides an interesting example as its level is strongly inversely associated with both WC and BMI and showed no response to weight loss but increased during weight maintenance in the WLWM cohort. Asparagine shares this change pattern with HDL cholesterol, thus representing a *profile of late change* after a weight loss and weight maintenance program, which is paralleled by the late appearance of improvement of glucose tolerance [7]. The increase in levels of asparagine after sustained weight loss was further validated in the replication cohort. Glycine, on the other hand, which was also strongly inversely associated with obesity, was not significantly affected during any part of the weight loss and weight maintenance program, which has also been observed in other weight loss studies [18, 21]. On the contrary, in the larger replication cohort, glycine increased after weight loss, which is expected compared to healthy individuals [8, 33]. In addition, cysteine has previously been associated with BMI, although since this AA was not identified in the MDC-CC, we could not test the association in this report, and the levels of cysteine did not respond to the intervention in the WLWM cohort [51]. Nevertheless, cysteine levels decreased in the larger replication cohort and further investigation on cysteine is encouraged. Variables including extent of weight loss, duration, pathogenesis, cohort size, and type of intervention could explain inconsistent changes in some AA levels. Furthermore, additional factors that potentially influence differences in AA levels independently of obesity include insulin resistance, adipose tissue distribution, and/or release of metabolites from the gut microbiota into the circulation [48, 52, 53].

For the purpose of patient risk-benefit assessment, we created different scores including classical risk factors (age, sex, AHT, SBP, and T2D status) and relevant AA levels. The OB-WC score was comprised of (in order of decreasing association strength) asparagine, tyrosine, alanine, glycine, valine, and glutamate, and the OB-BMI score of asparagine, tyrosine, valine, alanine, isoleucine, and glycine. The levels of both scores decreased after weight loss and weight maintenance. However, while two individuals may have an equal total AA association with obesity (i.e., equal OB scores), they may benefit differently from weight loss, since not all AAs respond equally to treatment. Therefore, we removed AAs that did not show long-term sustained change (towards levels found in healthy subjects) from the OB scores and created the WLWM-WC and WLWM-BMI scores defining treatment-modifiable association scores. Model predictions were calculated for both sets of scores. We found that for both the OB and the WLWM scores, WC had a slightly higher prediction value than BMI. Hypothetically, the weight loss and weight maintenance scores could be used in evaluating the treatment-specific likelihood of benefit for an individual, although this could not be validated in the replication cohort. We observed that both WC and BMI scores decrease substantially in individuals with ≥10% weight loss due to favorable changed amino acid levels and potentially, systolic blood pressure, while the scores increased in the <10% weight loss group due to unchanged or unfavorable change of amino acid levels and systolic blood pressure.

We also indirectly assessed the risk of future T2D [14] and CVD [15] by evaluating the effect of weight loss on the DM-AA score. The DM-AA score decreased during weight loss (both WLWM and replication cohort) and sustained during weight maintenance, indicating that the risk for T2D and CVD was reduced in the long-term by the weight loss and weight maintenance program. In addition, an increased risk was found in the group with <10% weight loss (replication cohort). When analyzing the whole cohort, we found a strong correlation between the change in BMI and the corresponding change in DM-AA score after weight loss. Since the DM-AA score, which consists of isoleucine, tyrosine, and phenylalanine, has been associated with insulin resistance, T2D, and cardiovascular risk, decrease of an elevated DM-AA score level may show decreased risk of developing these disorders [12, 14, 15, 19].

There are some limitations to this investigation that should be recognized. The three cohorts analyzed were not matched and the differences in, for example, age, gender distribution, and T2D, might affect the generalizability of the results. Even so, we want to highlight that the first study (MDC-CC) was used to find AAs associated with WC and BMI, which requires a larger cohort with a wider range of BMI motivating the use of a population-based approach. The weight loss and weight maintenance program was conducted in a small group of subjects that has the benefit of being run in a supervised clinical trial setting targeting obese individuals (separate from the MDC-CC); the replication cohort was included to further evaluate the changes observed after ≥10% weight loss as well as comparing results with subjects with <10% weight loss. Furthermore, nutritional information was considered neither in the WLWM nor in the replication cohort other than that the participants reduced their daily calorie intake, and nutritional content may influence the results. To achieve the main goal of weight loss and to maintain reduced weight, the overall calorie intake was restricted and unhealthy eating habits were corrected (with the help of a dietician and the behavioral change program); diet composition was not accounted for.

In conclusion, we investigated the association between circulating AA levels and abdominal or general obesity. From a panel of 18 AAs, eight consistently showed associations with obesity. Out of these eight AAs, five changed as a response to weight loss towards levels found in lean subjects and one during weight maintenance, underlining the importance of more research on weight maintenance after weight loss. The change of these six AAs was validated in a replication cohort. We developed scores based on WC or BMI including sex, age, SBP, AHT, T2D status, and independently associated AAs that describe the AA-associated burden of obesity and found that each risk score decreased with ≥10% weight loss. These scores may aid in the evaluation on an individual level as to who will benefit from weight loss treatment in terms of cardiometabolic risk. The concept of combining epidemiological data with response to intervention may be implemented in other areas of metabolic disease.

## Conflicts of Interest

The authors declare no conflicts of interest.

## Supplementary Material

The information of supplementary materials are as follows: Supplementary Figure 1: Logistic regression of amino acids associated with obesity. Supplementary Figure 2: PCA score plot (A) and OPLS-DA score plot (B) of metabolites analysed in the WLWM cohort. Supplementary FIgure 3: Change in BMI regressed on obesity-, WLWM- and diabetes-scores. Supplementary Table 1: Obesity and diabetes scores at Baseline, Follow-up and change from Baseline to Follow-up in the replication cohort.

## Figures and Tables

**Table 1:** Baseline characteristics of the participants in the Malmö Diet and Cancer Cardiovascular Cohort (MDC-CC), the Weight Loss and Weight Maintenance intervention study, and the replication cohort.

	MDC-CC	Weight Loss and Weight Maintenance intervention			Replication cohort	
		At baseline	After weight loss^a^	After weight maintenance^b^	At baseline	At follow-up^c^
*N*	804	12	12	12	83	83
Age (years)	59.2 ± 5.5	35.7 ± 12.3	35.8 ± 12.3	36.4 ± 12.3	44.5 ± 11.9	45.4 ± 11.9
Sex (% women)	51.3	75	75	75	67	67
Weight (kg)	77.8 ± 14.8	132.8 ± 23.2	110.7 ± 23.6^f^	106.4 ± 25.5^g^	123.5 ± 22.0	104.8 ± 23.1^i^
Weight loss (%)	—	—	−17.1 ± 5.6	−20.1 ± 10.2	**—**	−15.0 ± 11.0
BMI (kg/m^2^)	27.1 ± 4.5	44.2 ± 5.8	36.9 ± 6.6^f^	35.4 ± 7.3^g^	42.5 ± 5.8	36.1 ± 6.6^i^
Waist circumference (cm)	88.6 ± 13.8	130.6 ± 10.0^d^	112.5 ± 14.2^f^	107.7 ± 15.0^g^	125.6 ± 15.7	111.2 ± 17.7^i^
Fasting glucose (mmol/L)	5.4 ± 1.4	4.8 ± 0.6	4.6 ± 0.7	4.6 ± 0.3	6.2 ± 2.5	5.6 ± 1.7^i^
Fasting insulin (*μ*U/mL)	9.5 ± 9.6	10.8 ± 6.1^d^	4.7 ± 3.7^f^	6.3 ± 4.6^g^	14.4 ± 8.2	9.6 ± 7.2^i^
HOMA-IR	2.4 ± 3.0	2.3 ± 1.3^d^	1.0 ± 0.9^f^	1.3 ± 1.0^g^	4.1 ± 3.1	2.6 ± 2.4^i^
HOMA-IR decrease (%)	—	—	−58.6 ± 24.4	−49.4 ± 25.2	**—**	−28.1 ± 55
LDL (mmol/L)	4.3 ± 1.0	3.1 ± 0.9	2.8 ± 0.5^d^	2.9 ± 0.9	3.1 ± 0.9	2.9 ± 0.9^i^
HDL (mmol/L)	1.3 ± 0.3	1.1 ± 0.4	1.0 ± 0.2^d^	1.3 ± 0.3^h^	1.1 ± 0.3	1.1 ± 0.3
Triglycerides (mmol/L)	1.5 ± 0.7	1.4 ± 0.8	0.8 ± 0.3^d,f^	0.8 ± 0.5^g^	1.5 ± 1.2	1.1 ± 0.7^i^
Diabetes (%)	7.2	0	0	0	19.3	19.3
Current smoker (%)	30.3	16.7	16.7	16.7	21.5^∗^	21.5^∗^
SBP (mmHg)	147.4 ± 18.7	126.7 ± 16.5^e^	121.5 ± 9.2	117.5 ± 14.4	129 ± 14.8	122 ± 15.2^i^
AHT (%)	23.7	0	0	0	30.1	30.1
Hypertension (%)	77.1	0	0	0	49.4	41.0

^a,b,c,e^The weight loss period with low-calorie diet lasted 101 ± 26 days; the weight maintenance period with counseling lasted 167 ± 37 days. Average time span between baseline and follow-up in replication cohort was 345 ± 195 days. Data expressed as mean ± SD. ^d^Data missing from one person at that time. Data missing from two persons at that time.

^f^ *p* < 0.05 between baseline and weight loss (Weight Loss and Weight Maintenance intervention).

^g^ *p* < 0.05 between baseline and weight maintenance (Weight Loss and Weight Maintenance intervention).

^h^ *p* < 0.05 between weight loss weight maintenance (Weight Loss and Weight Maintenance intervention).

^i^ *p* < 0.05 between baseline and follow-up (replication cohort).

^∗^Smoke status is lacking for 4 subjects.

**Table 2:** Amino acids associated with waist circumference and BMI in the Malmö Diet and Cancer Cardiovascular Cohort.

Amino acids	Model 1 (adjusted for age and gender)				Model 2 (adjusted for age, gender, SBP, AHT, and T2D status)			
	*β*_WC_ (SE)	*p*	*β*_BMI_ (SE)	*p*	*β*_WC_ (SE)	*p*	*β*_BMI_ (SE)	*p*
Alanine	3.359 (0.393)	<0.001	1.215 (0.153)	<0.001	3.337 (0.377)	<0.001	1.197 (0.148)	<0.001
Arginine	−0.452 (0.408)	0.268	−0.241 (0.158)	0.128	−0.407 (0.392)	0.299	−0.220 (0.152)	0.149
Asparagine	−2.800 (0.397)	<0.001	−0.996 (0.155)	<0.001	−2.137 (0.391)	<0.001	−0.822 (0.153)	<0.001
Glutamate	2.642 (0.401)	<0.001	0.791 (0.157)	<0.001	2.125 (0.393)	<0.001	0.591 (0.154)	<0.001
Glutamine	−2.029 (0.409)	<0.001	−0.780 (0.159)	<0.001	−1.613 (0.398)	<0.001	−0.632 (0.155)	<0.001
Glycine	−3.348 (0.413)	<0.001	−1.286 (0.160)	<0.001	−2.940 (0.403)	<0.001	−1.138 (0.157)	<0.001
Isoleucine	2.746 (0.448)	<0.001	0.849 (0.175)	<0.001	2.054 (0.448)	<0.001	0.594 (0.175)	0.001
Leucine	1.377 (0.447)	0.002	0.431 (0.174)	0.013	0.681 (0.443)	0.124	0.180 (0.172)	0.295
Lysine	0.615 (0.408)	0.133	0.231(0.158)	0.144	0.450 (0.393)	0.252	0.173 (0.153)	0.259
Methionine	0.670 (0.427)	0.117	0.191 (0.166)	0.249	0.632 (0.410)	0.124	0.168 (0.160)	0.293
Ornithine	−1.184 (0.409)	0.004	−0.467 (0.159)	0.003	−1.245 (0.393)	0.002	−0.482 (0.153)	0.002
Phenylalanine	2.726 (0.408)	<0.001	1.006 (0.159)	<0.001	2.584 (0.392)	<0.001	0.951 (0.153)	<0.001
Proline	1.081 (0.414)	0.009	0.405 (0.161)	0.012	1.128 (0.397)	0.005	0.422 (0.155)	0.007
Serine	−1.566 (0.411)	<0.001	−0.599 (0.160)	<0.001	−0.969 (0.404)	0.017	−0.382 (0.157)	0.015
Threonine	−1.512 (0.406)	<0.001	−0.541 (0.158)	0.001	−1.106 (0.396)	0.005	−0.396 (0.154)	0.01
Tryptophan	0.434 (0.428)	0.311	0.097 (0.166)	0.558	0.330 (0.411)	0.423	0.052 (0.160)	0.743
Tyrosine	3.835 (0.387)	<0.001	1.367 (0.152)	<0.001	3.586 (0.376)	<0.001	1.261 (0.148)	<0.001
Valine	3.021 (0.418)	<0.001	1.085 (0.163)	<0.001	2.408 (0.418)	<0.001	0.863 (0.163)	<0.001
DM-AA score	4.091 (0.406)	<0.001	1.425 (0.159)	<0.001	3.648 (0.170)	<0.001	1.256 (0.156)	<0.001

Models 1 and 2: linear regression analysis to test amino acids association with waist circumference and BMI; *β*: standardized regression coefficient; SE: standard error.

**Table 3:** Backward elimination of amino acids associated with obesity traits in the Malmö Diet and Cancer Cardiovascular Cohort and limited to amino acids which improved with the Weight Loss and Weight Maintenance program.

Amino acids	Model 1 (AAs associated with obesity traits in MDC-CC)				Model 2 (improved AAs after WLWM intervention)			
	*β*_WC_	*p*	*β*_BMI_	*p*	*β*_WC_	*p*	*β*_BMI_	*p*
Alanine	2.39	<0.001	0.92	<0.001	2.56	<0.001	0.93	<0.001
Asparagine	−4.01	<0.001	−1.40	<0.001	−4.52	<0.001	−1.64	<0.001
Glutamate	0.96	0.008	—	0.131	1.04	0.003	—	—
Glycine	−1.41	<0.001	−0.58	<0.001	—	—	—	—
Isoleucine	—	0.36	−0.61	0.034	—	0.166	0.16	0.369
Phenylalanine	—	0.44	—	0.335	—	0.201	—	—
Tyrosine	2.96	<0.001	1.03	<0.001	3.56	<0.001	1.28	<0.001
Valine	1.21	0.005	1.01	<0.001	—	—	—	—
Sex	−13.4	<0.001	0.12	0.714	−14.8	<0.001	−0.08	0.794
Age	−0.12	0.068	−0.06	0.014	−0.15	0.017	−0.08	0.002
SBP	0.05	0.017	0.02	0.002	0.05	0.015	0.02	0.002
AHT	2.27	0.005	0.98	0.003	2.48	0.003	1.04	0.002
T2D status	4.26	0.002	1.50	0.007	5.27	<0.001	1.71	0.003

Model 1: amino acids with independent association with waist circumference to abdominal obesity and BMI to obesity, respectively. Regression coefficients from the backward elimination of amino acids associated with waist circumference and BMI in the Malmö Diet and Cancer Cardiovascular Cohort (*R*^2^_WC_ = 0.54 and *R*^2^_BMI_ = 0.31 for Model 1). Model 2: amino acids that improve with the Weight Loss and Weight Maintenance program (i.e., excluding glycine and valine based from Table 3), with independent association with waist circumference and abdominal obesity and BMI and obesity (*R*^2^_WC_ = 0.53 and *R*^2^_BMI_ = 0.28 for Model 2). Covariates; age, sex, SBP, AHT, and T2D status.

**Table 4:** Amino acid and amino acid score profiles during the Weight Loss and Weight Maintenance program.

Profile	Amino acids and scores	Uncorrected predictive loadings^a^		
		Weight loss	Weight maintenance	Whole course
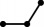	Asparagine	−0.042	**0.190**	**0.108**
	Glutamine	−0.029	**0.151**	**0.089**
	Methionine	−0.012	**0.170**	**0.140**
	Proline^b^	−0.077	**0.161**	0.050

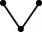	Lysine	−**0.095**	**0.144**	0.062
	Valine	−**0.170**	**0.160**	−0.087

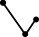	Alanine	−**0.189**	**0.188**	−**0.150**
	Aspartate	−**0.214**	**0.111**	−**0.233**
	Tyrosine	−**0.177**	**0.201**	−**0.077**
	Phenylalanine	−**0.201**	**0.205**	−**0.107**
	Tryptophan	−**0.191**	**0.132**	−**0.130**
	DM-AA	−**0.196**	**0.189**	−**0.167**
	OB-BMI	−**0.210**	**0.111**	−**0.231**

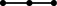	*β*-alanine	−0.049	−0.032	−0.079
	Cysteine	−0.045	0.008	−0.048
	Glycine	0.031	0.045	0.062
	Threonine	−0.041	0.083	0.018

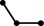	Glutamate	−**0.195**	−0.053	−**0.271**
	Isoleucine/ leucine	−**0.157**	0.046	−**0.191**
	WLWM-WC	−**0.208**	0.048	−**0.247**
	WLWM-BMI	−**0.198**	0.062	−**0.229**
	OB-WC	−**0.216**	0.072	−**0.253**
	Serine	−**0.229**	0.063	−**0.279**

^a^Loadings obtained from multivariate data analysis and values in bold face indicate a significant change. ^b^The profile for proline is not significance since the increase over the whole course is not significance. The profile for each metabolite describes the change in metabolite levels between the three time points: baseline, after weight loss and after weight maintenance. Each time point is represented by a dot.
