# Supplementary material for: Amino Acid Signatures to Evaluate the Beneficial Effects of Weight Loss
Source: Int J Endocrinol. 2017 Apr 16;2017:6490473. doi: 10.1155/2017/6490473 (PMC5412138; doi:10.1155/2017/6490473)
Supplement: Supplementary file 1 — The information of supplementary materials are as follows: Supplementary Figure 1: Logistic regression of amino acids associated with obesity. Supplementary Figure 2: PCA score plot (A) and OPLS-DA score plot (B) of metabolites analysed in the WLWM cohort. Supplementary FIgure 3: Change in BMI regressed on obesity-, WLWM- and diabetes-scores. Supplementary Table 1: Obesity and diabetes scores at Baseline, Follow-up and change from Baseline to Follow-up in the replication cohort. [file 6490473.f1.pdf]

Supplementary Figure 1

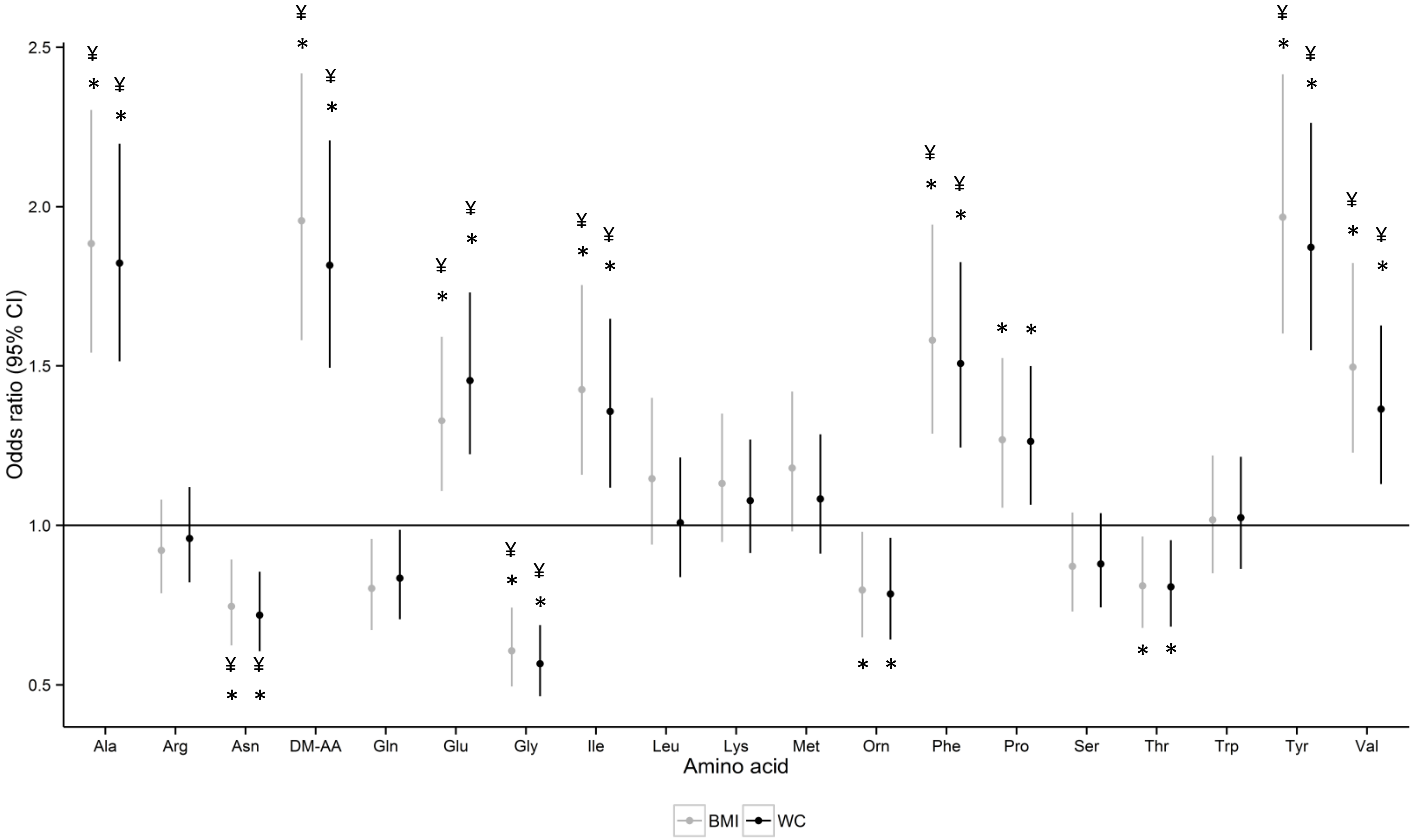

Amino acids associated with abdominal obesity, represented as BMI (grey bars) or waist circumference (WC, black bars), in the Malmö Diet and Cancer Cardiovascular Cohort. BMI was defined as ≥ 30 kg/m^2 and WC was defined as > 88 cm for women and > 102 cm for men, according to National Cholesterol Education Program. Analysis was adjusted for age, gender, SBP, AHT and T2D status. DM-AA; Diabetes-predictive amino acid score. *p<0.05, [¥]p-value significant after Bonferroni correction.
