# Supplementary material for: Amino Acid Signatures to Evaluate the Beneficial Effects of Weight Loss
Source: Int J Endocrinol. 2017 Apr 16;2017:6490473. doi: 10.1155/2017/6490473 (PMC5412138; doi:10.1155/2017/6490473)
Supplement: Supplementary file 2 [file 6490473.f2.pdf]

Supplementary Figure 2

A **B**

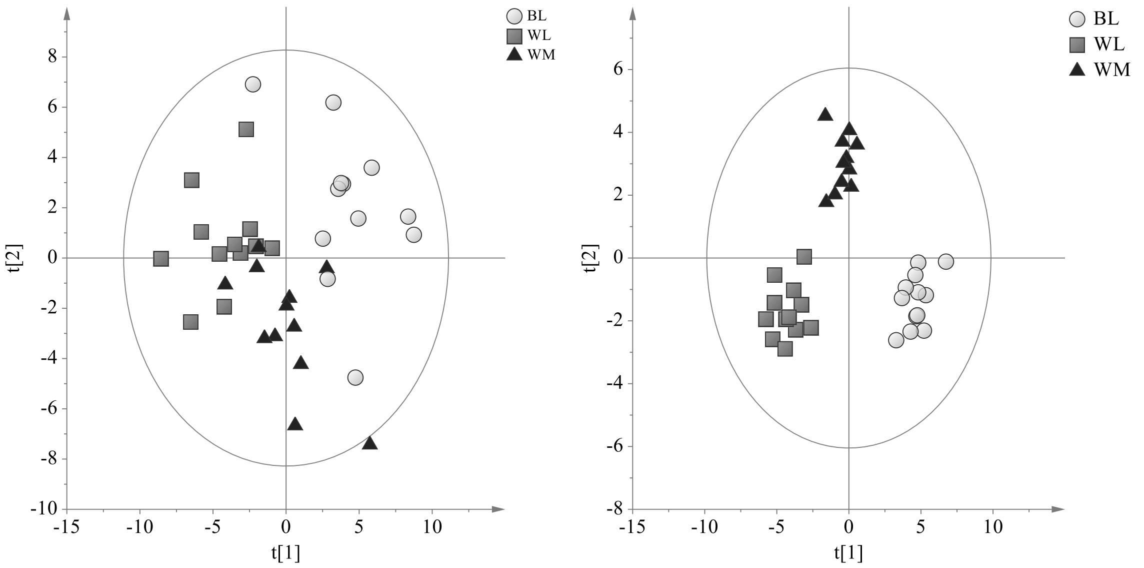

A principal component analysis (PCA) score plot summarizing the metabolite levels in all subjects at baseline (BL), after weight loss (WL) and after weight maintenance (WM) (A). The score vectors, t[1] and t[2], describes the largest and second largest amount of variance in the data. An orthogonal projection to latent structures discriminant analysis (OPLS-DA) score plot for all the three time-points, *i.e.* BL, WL and WM (B). The score vectors t[1] and t[2] describes the predictive variance between BL, WL and WM.
