# Supplementary material for: Amino Acid Signatures to Evaluate the Beneficial Effects of Weight Loss
Source: Int J Endocrinol. 2017 Apr 16;2017:6490473. doi: 10.1155/2017/6490473 (PMC5412138; doi:10.1155/2017/6490473)
Supplement: Supplementary file 3 [file 6490473.f3.pdf]

Supplementary Figure 3

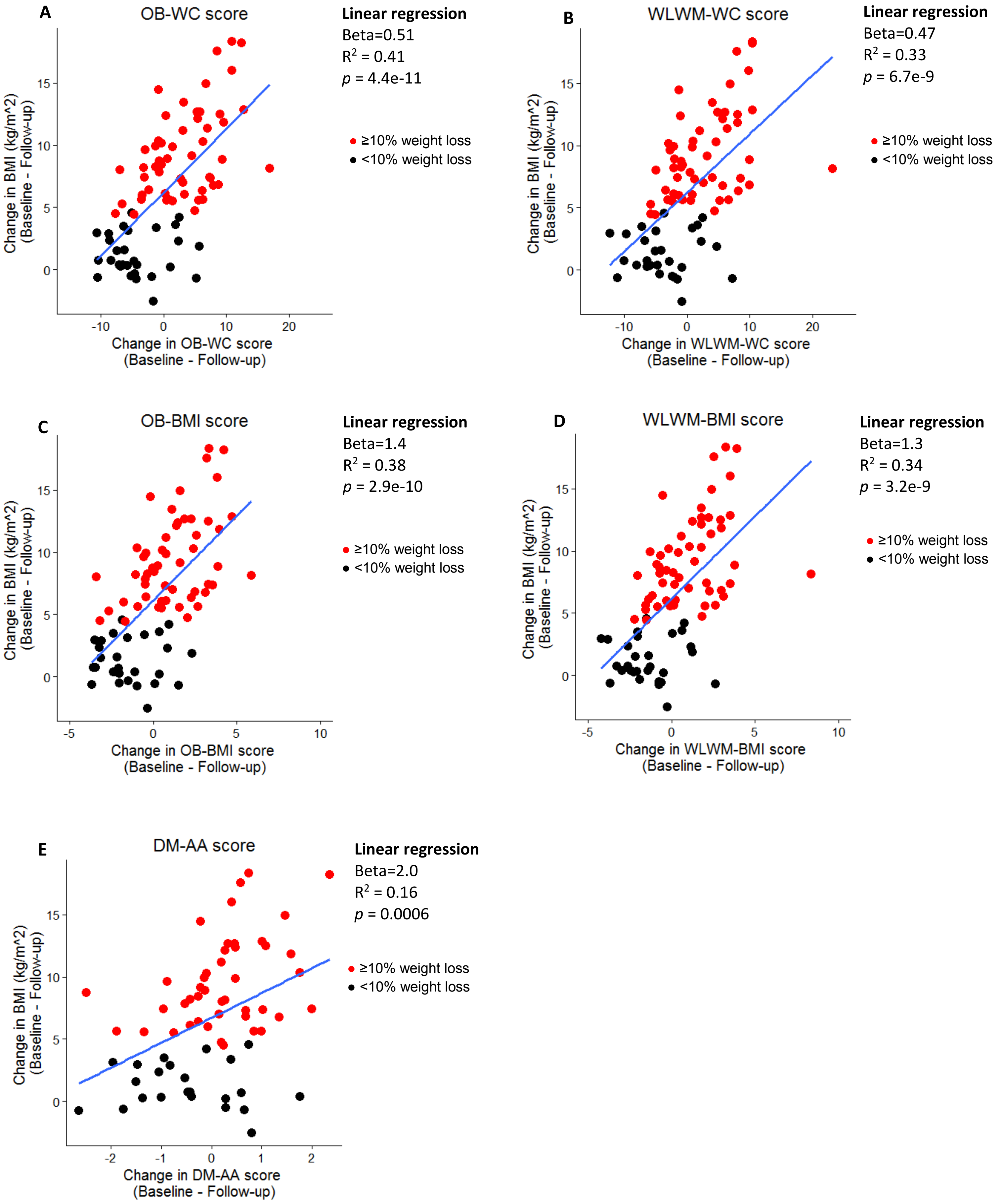

The association between change in BMI and change in OB-WC (A), WLWM-WC (B), OB-BMI (C), WLWM-BMI (D) and DM-AA scores (E). The correlation coefficient for change in BMI and change in OB-WC (R=0.63, $p<0.0001$), change in WLWM-WC (R=0.60, $p<0.0001$), change in OB-BMI (R=0.62, $p<0.0001$), change in WLWM-BMI (R=0.62, $p<0.0001$), and DM-AA score (R=0.41, $p=0.0006$). Spearman's rank correlation coefficient was used for each analysis. Individuals with T2D were excluded (n=17) when analyzing the DM-AA score.
