# Supplementary material for: Amino Acid Signatures to Evaluate the Beneficial Effects of Weight Loss
Source: Int J Endocrinol. 2017 Apr 16;2017:6490473. doi: 10.1155/2017/6490473 (PMC5412138; doi:10.1155/2017/6490473)
Supplement: Supplementary file 4 [file 6490473.f4.pdf]

Supplementalary Table 1 Obesity and diabetes scores at Baseline, Follow-up and change from Baseline to Follow-up in the replication cohort.

	**Score**	**Baseline**	**Follow-up**	**Change from baseline to follow-up**
	OB-WC	88.3 (11.4)	87.7 (11.6)	0.54 (6.22)
	WLWM-WC	88.9 (11.3)	88.4 (11.6)	0.52 (6.11)
**All participants**	OB-BMI	27.5 (2.77)	27.3 (2.63)	0.24 (2.26)
	WLWM-BMI	27.4 (2.59)	27.2 (2.57)	0.22 (2.21)
	DM-AA	-2.24e^{-17} (1)	-2.54e^{-17} (1)	-2.05e^{-18} (1.05)
	OB-WC	88.2 (11.5)	85.1 (11.2)	3.09 (5.46)***
	WLWM-WC	88.7 (11.5)	85.9 (11.6)	2.79 (5.57)**
**≥10% Weight loss**	OB-BMI	27.6 (2.68)	26.5 (2.25)	1.15 (2.01)***
	WLWM-BMI	27.5 (2.49)	26.5 (2.32)	1.08 (2.00)***
	DM-AA	-0.007 (0.88)	-0.26 (0.95)	0.25 (0.96)*
	OB-WC	88.5 (11.3)	92.7 (11.7)	-4.23 (4.58)****
	WLWM-WC	89.2 (11.1)	92.9 (10.1)	-3.70 (4.69)***
**<10% Weight loss**	OB-BMI	27.2 (2.97)	28.7 (2.71)	-1.46 (1.65)***
	WLWM-BMI	27.1 (2.79)	28.5 (2.53)	-1.37 (1.65)***
	DM-AA	0.01 (1.22)	0.49 (0.92)	-0.47 (1.07)*

No significance difference between baseline and follow-up in the group with all participants. Data presented as mean (SD). Type 2 diabetes cases were excluded (n=17) when calculating DM-AA score. Difference between <10% and >10% weight loss for respective change in score from baseline to follow-up; OB-WC (p = 1.89e^{-7}), WLWM-WC (p = 1.92e^{-6}), OB-BMI (p = 4.25e^{-7}), WLWM-BMI (p = 6.65e^{-7}), and DM-AA (p = 0.0088). Significance cut-offs were as follows; *$p<0.05$, **$p<0.01$, ***$p<0.001$, ****$p<0.0001$.
